# Mineral wealth paradox: health challenges and environmental risks in African resource-rich areas

**DOI:** 10.1186/s12889-024-18137-1

**Published:** 2024-03-06

**Authors:** Emmanuel Adu Sarfo, Rabbi Tweneboah

**Affiliations:** 1 Faculty of Economic Sciences, National Research University Higher School of Economics, 20 Myasnitskaya Street, 101000 Moscow, Russia; 2 Department of Mathematics and Statistics, Youngstown State University, 1 University Plaza, OH 44555 Ohio, USA

**Keywords:** Mineral deposit, Onshore petroleum field, Environmental health risks, In-migration, Mining activities, Violent events, O15, Q3, Q24, Q25

## Abstract

**Background:**

Africa is blessed with vast arable land and enriched with valuable natural resources encompassing both renewable (like water, forests, and fisheries) and non-renewable (such as minerals, coal, gas, and oil). Under the right conditions, a natural resource boom should serve as an important driver for growth, development, and the transition from cottage industry to factory output. However, despite its wealth, Africa is often associated with the notion of a resource curse. Negative outcomes are often linked with mineral wealth. This paper investigates the causes of adverse health outcomes in resource-rich regions. The study provides empirical support for the natural resource curse with particular emphasis on the environmental health risks in Africa. We explore the multifaceted connections among mineral deposits, environmental risks, conflict events and population dynamics, shedding light on the complexities of resource-rich areas.

**Results:**

We amalgamate georeferenced data pertaining to 22 specific mineral deposits with information on the prevalence of reliance on compromised infrastructures at a spatial resolution of 0.5$^{\circ } \times 0.5^{\circ }$ for all of Africa between 2000 and 2017. Through comprehensive econometric analysis of environmental health risk factors, including reliance on contaminated water sources, open defecation, unimproved sanitation, particulate matter concentration, and carbon concentration, we uncover the intricate pathways through which mineral deposits impact public health. Our findings revealed the significant role of in-migration in mediating environmental health risks. Moreover, we found that the activities of extractive companies amplify certain environmental risks including reliance on unimproved sanitation and practices and particulate matter concentration. Conflict events emerge as a key mediator across all environmental health risks, underlining the far-reaching consequences of instability and violence on both local communities and the environment.

**Conclusion:**

The study contributes to the discourse on sustainable development by unraveling the nuanced associations between mineral wealth and health challenges. By drawing attention to the intricate web of factors at play, we provide a foundation for targeted interventions that address the unique environmental and health challenges faced by mineral-rich communities.

**Supplementary Information:**

The online version contains supplementary material available at 10.1186/s12889-024-18137-1.

## Introduction

The demand for large-scale extraction of mineral and metal resources in Africa continues to rise, driven in part by the global shift towards a low-carbon future [1]. According to the report of the United Nations Environment Programme (UNEP) [2], Africa holds a significant share of the world’s mineral reserves, approximately 30% located on the continent. Additionally, Africa holds around 8% of the world’s natural gas reserves and 12% of oil reserves, making it a crucial player in meeting the rising global demand for minerals, metals, oil, and natural gas. Currently, Africa hosts over 2000 industrial mining projects and many more planned.^1^ The continent’s rich resources present both opportunities and challenges.

Studies have offered insight into the economic consequences of mining [4]. Using night light as a measure of economic development, Mamo et al. [5] found that mining districts benefit from higher living standards relative to other districts in Africa. That is, through job opportunities (particularly in non-agriculture sectors; [6]) and investments in community development, mining may significantly contribute to poverty reduction and raising living standards. Kotsadam and Tolonen [7] documented that mining has the potential to enhance non-agricultural sectors, and provide cash-earning opportunities - including women of reproductive age [8]. Moreover, it has been shown that mineral wealth can spur infrastructure development, including the building of schools, hospitals, and road networks, as well as improve housing conditions, including proper sanitation and safe water [9, 10]. These improved conditions in turn reduce the incidence of environmental-related diseases such as respiratory infections [11], diarrhoea diseases [12], malaria, and undernutrition [8].

Although the aforementioned studies clearly outline the beneficial effects of mineral wealth and mining activities on economic and health outcomes, several other studies have reported adverse effects on health outcomes [13]. Direct environmental health effects are extensively researched at the community level. For instance, a study of Goan communities in India found that the production of iron ore had a detrimental influence on health outcomes due to fugitive dust emissions and consequent poor air quality [14]. It is recognized that mineral-rich communities can be impacted by a wide range of factors, such as transient populations, economic instability, or land degradation caused by the influx of mining activities [14–16]. Consequently, resulting in food insecurity [17], increased competition on water infrastructure [18], stunting growth in children [19], and other health-related outcomes [20]. The broad strand of literature assessing the impact of mineral wealth on health outcomes is mixed. Studies either identify positive, negative, or mixed effects of mining on a stretch of health outcomes in resource-rich regions [16, 17, 19]. It has been reported that mineral mining activities in developing countries had mixed effects on local communities; that is, there is a trade-off between wealth gain and health cost [21]. On one hand, they found that the presence of mines led to economic spillover effects, such as job creation and increased wages, which contributed to improved living standards for some households. On the other hand, their study noted that mineral mining led to a decline in local good health outcomes, particularly in terms of child mortality rates.

Our study aimed to understand the impact of mineral wealth on health outcomes by exploring the potential mechanisms that contribute to the observed negative effects on health. We argued that the observed adverse effect of mineral wealth on health [13, 21] can largely be attributed to two main factors; namely, ambient air quality and infrastructure strain.

First, the prevalence of artisanal mining has been shown to be high in mineral-rich areas [22], which is often accompanied by detrimental effects such as environmental degradation, hazardous working conditions, inadequate health and safety precautions, the involvement of child labor, and engagement in illegal mining operations. A growing body of studies suggests that mining sectors are connected to emissions of nitrogen oxide (NOx), sulfur oxide (SOx), and particulate matter (PM10 and PM2.5) from various activities of mine and greenhouse gas emission [23–25]. Leili and colleagues argued that these airborne particles are adversely affecting health by contributing to illness [26]: for instance, damaging the lungs, damaging the respiratory tract, and causing skin diseases by seeping into the skin both at the sites and in close residential areas. Also, climate change brought on by carbon emissions poses unprecedented challenges to global society in the form of extreme weather, sea level rise, infectious diseases, biodiversity loss, and food shortages [27]. Thereby contributing to negative health outcomes such as stunting and diarrheal diseases [19].

Second, natural resource extraction has been linked to urbanizing population agglomerations (mineralized urbanization)^2^ due to migration, shifting sectoral job patterns, improved housing, and higher domestic investment [18, 29]. Prior studies demonstrate that societies in Africa (such as West African countries) fought over territories that had rich-minerals even before the arrival of the European as these resources signified embodiment of power and wealth [30, 31]. The the arrival of the European and the development of mining industry led to a windfall in infrastructural development in these areas [32], ultimately increasing population density. However, it has been reported that the high influx of job seekers to remote and rural mining areas can exacerbate the pressure on available water resources and infrastructures [33]. Additionally, through geospatial visualization, Leuenberger et al. [18] highlights the spatial distribution of water sources, infrastructure, and health facilities in relation to the mining areas. They identified potential hotspots of water scarcity, contamination, and inadequate sanitation facilities, which can significantly impact the health and well-being of the communities living and working in these mining settings. We argue that the rapid in-migration of individuals to mining areas, driven by the lure of potential economic prosperity, may outpace the rate of local development, thereby wearing down existing public amenities and intensifying competition for their use. This increases the risk of reliance on compromised infrastructure, including water and sanitation facilities, plausibly leading to health risks and negative outcomes. Furthermore, natural resources frequently serve as catalysts for internal conflicts, as various factions vie for dominion over these resources or utilize them to fund their hostile endeavors. A growing body of empirical evidence suggests that rents on primary commodities and natural resources, particularly oil and other point-source resources, increase the likelihood of civil conflicts and wars in Africa by undermining the state or funding rebels, sometimes even by multinational corporations [34–37]. Several studies have reported that governments in many resource-rich countries are unable to safeguard basic security for their citizens since the wealth of resources elicits violence, theft, and looting often financed by rebels and competing warlords [38, 39]. These events often result in the displacement of lives and properties, straining existing basic infrastructures.

The study’s distinctiveness lies in its investigation of the roots of the observed poor health outcomes in mineral-rich areas, transcending beyond the impact of mining activities. With few exceptions, prior studies have attributed the adverse health outcomes in mineral-rich areas solely to activities by extractive industries. The study further contributes to the limited understanding of the impact of mining activities on ambient air quality in the developing world. It contributes to the literature on the impact of population dynamics on health outcomes by highlighting one of its consequences on available amenities (consequently, increasing the risk of reliance on compromised structures).

This study explored the possible mechanisms that lead to the observed adverse effects of mineral wealth on health outcomes in Africa. The findings could provide new insights for policymakers and various stakeholders to design or adjust schemes related to reducing environmental pollution and health-related risk associated with the extraction of these resources.

## Methods

### Data sources and management

This layer of the section presents a brief description of the study’s main variables of interest. The outcome variables highlighted includes reliance on contaminated water source, open defecation, and unimproved sanitation, as well as $CO_{2}$ and *PM*2.5 concentration. The main explanatory variable highlighted here includes mineral deposit and onshore petroleum fields.

#### Outcome variables

Reliance on contaminated water measures the percentage of the population without access to a clean drinking water source at each 5x5 km pixel. Examples of clean drinking water sources may include piped water, boreholes, wells, protected springs, or other improved water sources. The coverage percentage indicates the proportion of people in the given geographic area who have access to the specified type of drinking water. Reliance on Open defecation and other Unimproved Sanitation represents the percentage of the population without access to a specific type of sanitation facility at each 5x5 km pixel. Sanitation facilities may include flush toilets, pit latrines, composting toilets, or other improved sanitation options. The coverage percentage indicates the proportion of people in the given geographic area who have access to the specified type of sanitation facility. The original data was taken from Institute for Health Metrics and Evaluation (IHME) [40].^3^ We created a grid cell of size 50km $\times$ 50km across the entire African continent and compute for each grid cell the average of our outcome variables from 2000 to 2017.

The aforementioned variables were chosen for our study as it allowed us to measure the level of competition/unavailability of crucial amenities (infrastructure strain) in a locality. For instance, open defecation is more common in places where there is limited or increased competition on sanitation facilities such as flush toilets, pit latrines, and composting toilets. Moreover, the use of these variables enables our study to provide intuitive explanation for the several empirical evidence that documented adverse health outcomes in resource-rich regions [13, 21]. The selected IHME variables, which measure reliance on compromised structures, are considered a good metric for evaluating the disease burden attributable to unsafe drinking water, sanitation, and hygiene at the local level [41–43].

We additionally use two outcomes to capture the risk factor within a grid cell. The variable $CO_2$ Concentration represents the mean concentration of carbon dioxide ($CO_2$) contained within a vertical column of dry air that extends from the Earth’s surface up to the outer boundary of the atmosphere. This concentration measurement is typically expressed in parts per million (ppm) by volume. The data used for this variable are derived from NASA’s Orbiting Carbon Observatory-2 (OCO-2) project. The provided raster dataset captures this $CO_2$ concentration by utilizing a 10-kilometer grid. The process involves aggregating one year’s worth of $CO_2$ concentration data and then utilizing linear interpolation techniques to address any gaps or missing values within the dataset. The underlying data that contribute to this variable’s computation are sourced from the OCO-2 Science Team/Michael Gunson, Annmarie Eldering [44] project. This variable serves as a critical indicator of atmospheric $CO_2$ levels, providing insights into the distribution and spatial patterns of $CO_2$ across Africa. As mentioned earlier, this variable is of particular importance to understand how mineral wealth leads to the deterioration of the climate and vegetation. Mineral-rich regions have been noted as experiencing rising temperatures, changes in precipitation, and rising sea level as a result of rising $CO_2$ emissions [45]. As such we’re able to directly link our results to the observed negative health outcomes (such as stunting and wasting in children) documented in the literature

The variable Particulate Matter (PM2.5) Concentration represents the average concentration of fine particulate matter (PM2.5) present in the ambient air. PM2.5 refers to particles suspended in the air with a diameter of 2.5 micrometers or smaller, which can pose health risks when inhaled due to their ability to penetrate deep into the respiratory system. This variable is of critical importance in understanding the impact of ambient air pollution on global health. Data is available from 1990 to 2013 and is sourced from Brauer et al. [46].

#### Main independent variables

Our main variable of interest, Mineral Deposits in Africa, pertains to the spatial distribution of mineral resource deposits across the African continent. These mineral deposits encompass 22 specific minerals or mineral commodities that are considered critical for the economic and security interests of the United States, as of the year 2017^4^. The dataset consists of both point and polygon layers within a geodatabase from Schulz et al. [47].

An additional variable we use to capture mineral deposits across Africa is the number of petroleum onshore fields in a grid cell. The Petroleum Dataset (PETRODATA) is a comprehensive collection of global oil and gas fields. This dataset is specifically curated to provide detailed information about the geographic distribution of hydrocarbon reserves around the world. It is tailored for utilization within Geographic Information Systems (GIS), offering a specialized resource for visualization, manipulation, and in-depth analysis. Within this dataset, essential details about oil and gas fields are meticulously documented. This includes precise information about the geographical coordinates of these hydrocarbon reserves, enabling accurate spatial representation. Designed with GIS applications in mind, the dataset’s structure facilitates seamless integration into GIS platforms, allowing users to explore and interact with the data geographically. The data is taken from Lujala et al. [48].

### Study design and statistical analysis

The cross-sectional design was used to achieve the study’s objectives. The goal of the study is to expand current understanding of the intricate relationship between resource endowment and environmental health concerns in Africa. The ordinary least square (OLS) technique is used to estimate the following reduced-form specification:

1 $$\begin{aligned} \text{ H}_{i} = \beta_{0}+\beta_{1} {mineral}_{i} +\textbf{X}_{i}^{\prime } \varvec{\Psi } + \Upsilon_{c} + \varepsilon_{i} \end{aligned}$$

where; $\text{ H}_{i}$ represents the percentage of people relying on either open defecation, contaminated water, or unimproved sanitation in grid cell *i*, It also refers to the amount of carbon concentration or particle concentration in grid cell *i*. Moreover, ${mineral}_{i}$ represents the number of mineral deposits or petroleum onshores in grid *i*.^5^$\textbf{X}_{i}^{\prime }$ is a set of grid cell level covariates that might confound the relationship between mineral deposits or petroleum onshore sites and Environmental Health Risk Factors, including geographical and climatic covariates. $\Upsilon_{c}$ represents the country fixed effects, $\beta_{1}$ is the coefficient on the linear term of a mineral deposit or petroleum onshore, and it is expected to be positive, indicating a positive association between mineral deposits or petroleum onshore sites and Environmental Health Risk Factors. $\varepsilon_{i}$ is a grid cell-specific disturbance term. Country fixed effects are included with the purpose of controlling for time-invariant country-specific factors that could potentially codetermine the presence of mineral deposits or onshore petroleum field and reliance on poor structures: for example, economic development (poor states) and stringent environmental policies. Clustering was applied at the grid level to account for spatial variations and investigate spatial patterns or dependencies within individual grid cells.

The primary parameter of interest is $\beta_{1}$ which highlights the marginal impact mineral deposit has on the various environmental health risk variables. In contrast to the study by Berman et al. [34], which measured mineral deposits based on whether a mine is operational or closed, a situation that could introduce potential endogeneity concerns, our measurement of mineral deposits is not contingent upon such conditions. Consequently, it is plausibly exogenous.

We contend that economic, social, and health characteristics in a given region have little to no influence on the composition of mineral wealth in that region. As a result, the possibility of reverse causality is eliminated. The potential confounding factors could be rooted in geographic, climatic, environmental, and, in some extreme cases, economic factors. These probable confounders, however, have been taken into account. Based on the aforementioned arguments, our results can be given causal interpretation.

## Results

This section of the paper provides an overview of the main results, indicating that the existence of natural resources has had a substantial and statistically significant influence on environmental health risks over the past two decades. The presentation starts by discussing the outcomes of the baseline cross-country regression models, which examines the reliance on unimproved infrastructure from 2000-2017. Additionally, the analysis reveals that the presence of natural resource not only contribute to increased reliance on unimproved structures but also influenced ambient air quality and climatic conditions. The section concludes by illustrating the pathways through which natural resources affect environmental health risk factors.

### The infrastructure strain effects - baseline results

Tables 1, 2 and 3 report the baseline results of the infrastructure strain impact owing to natural resource availability. Column (1) presents the unconditional regression result on the effect of our main regressors (i.e. mineral deposit and petroleum onshore sites) on the outcome variables (i.e. reliance on either contaminated water, open defecation, or unimproved sanitation); respectively. We progressively included a variety of geographic factors, climatic factors, and economic growth in the specification in Columns (2), (3), and (4). In all specifications of the aforementioned Tables, mineral deposit, and petroleum onshore field, our coefficients of interest, are positive and significant at the 1% level. Country fixed effects and clustering at the grid-cell level were incorporated in all specifications (See Methods section for details).

#### Reliance on contaminated water

Based on results in Column 1 of Table 1, the unconditional regression analysis indicates a positive association between the presence of mineral resources and the extent to which individuals rely on contaminated water. That is, a unit increase in mineral deposits and the presence of petroleum fields within a given 50km grid area corresponds to a 33.6% and 16.5% increase in reliance on polluted water sources, respectively. This relationship is statistically significant at the 1% level. In Column 2, the statistical significance of the mineral deposit and petroleum field variables persisted even after controlling for the potential influence of geographic location and elevation characteristics.

**Table 1:** Natural Resource and reliance on contaminated water: baseline results

	(1)	(2)	(3)	(4)
Mineral deposit	0.3364^a^	0.3474^a^	0.2124^a^	0.1903^a^
	[0.0416]	[0.0457]	[0.0295]	[0.0302]
Petroleum field	0.1654^a^	0.2768^a^	0.0632^c^	0.0436
	[0.0341]	[0.0382]	[0.0339]	[0.0341]
Latitude		-0.0585^a^	-0.0460^a^	-0.0471^a^
		[0.0055]	[0.0051]	[0.0051]
Longitude		-0.1350^a^	-0.0875^a^	-0.0889^a^
		[0.0067]	[0.0053]	[0.0053]
Mean elevation		0.0009^a^	0.0012^a^	0.0012^a^
		[0.0001]	[0.0001]	[0.0001]
Ln(distance to coast)		-0.1582^a^	-0.4702^a^	-0.4613^a^
		[0.0237]	[0.0251]	[0.0250]
Temperature			0.1343^a^	0.1383^a^
			[0.0137]	[0.0137]
Precipitation			0.0203^a^	0.0204^a^
			[0.0012]	[0.0012]
Vegetation			0.0009^a^	0.0009^a^
			[0.0000]	[0.0000]
Night luminosity				0.1353^a^
				[0.0490]
Observations	12,529	12,428	12,356	12,356
R-squared	0.6645	0.6873	0.7765	0.7770
Country FE	Yes	Yes	Yes	Yes
Clustering var.	Grid Cell	Grid Cell	Grid Cell	Grid Cell

The table presents the coefficient estimates of the OLS regression with multiple fixed effects, which examines the impact of mineral deposits and petroleum fields on reliance on contaminated water sources. We define all variables in Appendix A. In columns (1) to (4), we include country-fixed effects, and all variables are clustered at the grid level. Heteroscedasticity-robust standard errors are reported in parentheses. ^a^, ^b^, and ^c^ indicate statistical significance at the 1%, 5%, and 10% levels (two-tailed), respectively

The results in column 3 incorporate both geographic and climatic covariates. The statistical significance and economic impact of the mineral deposit variable remained unchanged but the statistical significance of the onshore petroleum field was at the 10% level. The coefficient of interest indicates that within a specific grid cell, a unit increase in mineral deposit and petroleum field is associated with a 21.4% and 6.3% increase in reliance on contaminated water; respectively, conditional on a set of geographic and climatic features. In Column 4, the statistical significance of the mineral deposit remained strong at the 1% level. However, the significance of the onshore petroleum field diminished after accounting for night light in the analysis. Specifically, a one-unit increase in mineral deposits within a given grid area is related to an increase in the reliance on contaminated sources by 19%, conditional on a set of economic growth and geo-climatic factors.

#### Reliance on open defecation

Table 2 presents the baseline results of the impact of natural resources on individuals’ reliance on open defecation (a health risk behavior). Based on the results presented in Column 1, there is a positive relationship observed between the presence of mineral deposits/onshore petroleum fields and the level of reliance on open defecation. Specifically, a unit increase in mineral deposit (petroleum field) results in a 36.4%(34.8%) increase in the level of reliance on open defecation in a given cell grid; respectively. The associations are statistically significant at the 1% level.

**Table 2:** Natural resources and reliance on open defecation: baseline results

	(1)	(2)	(3)	(4)
Mineral deposit	0.3642^a^	0.3876^a^	0.2557^a^	0.1905^a^
	[0.0377]	[0.0417]	[0.0278]	[0.0313]
Petroleum field	0.3485^a^	0.4421^a^	0.2438^a^	0.1861^a^
	[0.0398]	[0.0432]	[0.0377]	[0.0366]
Latitude		-0.0554^a^	-0.0455^a^	-0.0485^a^
		[0.0056]	[0.0053]	[0.0053]
Longitude		-0.1424^a^	-0.0951^a^	-0.0993^a^
		[0.0070]	[0.0055]	[0.0055]
Mean elevation		0.0006^a^	0.0011^a^	0.0012^a^
		[0.0001]	[0.0001]	[0.0001]
Ln(distance to coast)		-0.1217^a^	-0.4789^a^	-0.4527^a^
		[0.0238]	[0.0269]	[0.0267]
Temperature			0.1657^a^	0.1775^a^
			[0.0160]	[0.0159]
Precipitation			0.0162^a^	0.0165^a^
			[0.0012]	[0.0012]
Vegetation			0.0010^a^	0.0009^a^
			[0.0000]	[0.0000]
Night luminosity				0.3992^a^
				[0.0664]
Observations	12,529	12,428	12,356	12,356
R-squared	0.6219	0.6437	0.7283	0.7329
Country FE	Yes	Yes	Yes	Yes
Clustering var.	Grid Cell	Grid Cell	Grid Cell	Grid Cell

The table presents the coefficient estimates of the OLS regression with multiple fixed effects, which examines the impact of mineral deposits and petroleum fields on reliance on open defecation. We define all variables in Appendix A. In columns (1) to (4), we include country-fixed effects, and all variables are clustered at the grid level. Heteroscedasticity-robust standard errors are reported in parentheses. ^a^, ^b^, and ^c^ indicate statistical significance at the 1%, 5%, and 10% levels (two-tailed), respectively

The preceding results remained qualitatively significant after controlling for geographic covariates in Column 2. Also, reassuringly, the coefficients of interest remained positively significant at the 1% level after accounting for both geographic and climatic factors in Column 3. Specifically, an increase of one unit in mineral deposit (onshore petroleum field) within a given 50km grid area is associated with a 25.6% (24.4%) increase in the level of reliance on open defecation, conditioned on a set of geographic and climatic factors; correspondingly. After controlling for a complete set of geo-climatic and economic factors, the coefficients of interest (i.e. mineral deposit and onshore petroleum field) remained statistically and economically significant at the 1%. Suggesting a high prevalence of health risk behavior (in this case open defecation) in resource-rich areas.

#### Reliance on unimproved sanitation

As mentioned earlier, Table 3 is structured similarly to Tables 1 and 2. Here, the dependent variable is unimproved sanitation. The results from the unconditional regression in Column 1 of Table 3 demonstrate a positive association between mineral deposits (onshore petroleum field) and the level of reliance on unimproved sanitation. Both of these relationships are statistically significant at the 1% level, indicating a clear and robust connection between mineral deposits, onshore petroleum fields, and the reliance on unimproved sanitation. That is, in a given grid cell, a unit increase in mineral deposit (onshore petroleum field) is associated with a 42.7% (33%) increase in the level of reliance on unimproved sanitation; respectively.

**Table 3:** Natural resources and reliance on unimproved sanitation: baseline results

	(1)	(2)	(3)	(4)
Mineral deposit	0.4271^a^	0.4226^a^	0.2880^a^	0.2166^a^
	[0.0414]	[0.0437]	[0.0282]	[0.0320]
Petroleum field	0.3303^a^	0.4162^a^	0.1820^a^	0.1188^a^
	[0.0433]	[0.0463]	[0.0412]	[0.0404]
Latitude		-0.0530^a^	-0.0394^a^	-0.0427^a^
		[0.0054]	[0.0049]	[0.0049]
Longitude		-0.1083^a^	-0.0651^a^	-0.0696^a^
		[0.0066]	[0.0052]	[0.0051]
Mean elevation		0.0010^a^	0.0011^a^	0.0012^a^
		[0.0001]	[0.0001]	[0.0001]
Ln(distance to coast)		-0.1998^a^	-0.5107^a^	-0.4820^a^
		[0.0257]	[0.0279]	[0.0275]
Temperature			0.0987^a^	0.1116^a^
			[0.0137]	[0.0136]
Precipitation			0.0183^a^	0.0187^a^
			[0.0012]	[0.0012]
Vegetation			0.0010^a^	0.0009^a^
			[0.0000]	[0.0000]
Night luminosity				0.4372^a^
				[0.0780]
Observations	12,529	12,428	12,356	12,356
R-squared	0.6485	0.6690	0.7613	0.7670
Country FE	Yes	Yes	Yes	Yes
Clustering var.	Grid Cell	Grid Cell	Grid Cell	Grid Cell

The table presents the coefficient estimates of the OLS regression with multiple fixed effects, which examines the impact of mineral deposits and petroleum fields on reliance on unimproved sanitation. We define all variables in Appendix A. In columns (1) to (4), we include country-fixed effects, and all variables are clustered at the grid level. Heteroscedasticity-robust standard errors are reported in parentheses. ^a^, ^b^, and ^c^ indicate statistical significance at the 1%, 5%, and 10% levels (two-tailed), respectively

In Column 2, we accounted for a set of geographic covariates, reassuringly, the statistical and economic significance remained unchanged. Similarly, the results remained qualitatively significant at the 1% level when a set of geo-climatic conditions were introduced to the specification in Column 3. After controlling for a complete set of both geo-climatic and economic factors in Column 4, the coefficients of interest changed quantitatively but remained unchanged qualitatively. That is, in a given grid cell, a unit increase in mineral deposit (onshore petroleum field) is associated with a 21.6% (11.8%) increase in the level of reliance on unimproved sanitation, conditioned on a set of geographic, climatic, and economic factors; respectively. These findings highlight the substantial impact that mineral deposits and onshore petroleum fields have on the prevalence of unimproved sanitation practices within specific geographic areas.

### The ambient air quality effect - baseline results

The baseline findings addressing the effect of natural resources on air quality are shown in Tables 4 and 5. In these tables, Column (1) displays the unconditional regression results for our main variables of interest, namely mineral deposit and petroleum onshore sites, on the respective outcome variables (i.e. particulate matter concentration and $CO_2$ concentration). Subsequently, we gradually introduce various geographic and climatic factors in Columns (2), (3), and (4) to further specify the models. To account for time-invariant country-specific characteristics that might influence the existence of mineral reserves or mining practices and ambient air quality, we integrated country-fixed effects.

#### Particulate matter concentration

Table 4 presents the findings on the influence of natural resource endowment on particulate matter concentration (PM). Based on the results provided in Column 1, the unconditional regression results showed a positive and significant relationship between mineral deposits (onshore petroleum field) and PM concentration. Specifically, a one-unit increase in mineral deposits (number of onshore petroleum fields) leads to a 0.6349 (0.6886)-point increase in the concentration of particulate matter within a given 50km grid area. This relationship is significant at the 1% level.

**Table 4:** Natural resources and particulate matter concentration: baseline results

	(1)	(2)	(3)	(4)
Mineral deposit	0.6349^a^	0.6219^a^	0.5932^a^	0.2583^a^
	[0.1405]	[0.1406]	[0.1404]	[0.0654]
Petroleum field	0.6886^a^	0.5708^a^	0.4314^a^	0.1349^a^
	[0.0868]	[0.0769]	[0.0675]	[0.0383]
Latitude		0.0167^a^	0.0218^a^	0.0063^b^
		[0.0043]	[0.0041]	[0.0026]
Longitude		0.0204^a^	0.0239^a^	0.0027
		[0.0057]	[0.0050]	[0.0036]
Mean elevation		0.0000	-0.0004^a^	-0.0000
		[0.0000]	[0.0001]	[0.0001]
Ln(distance to coast)		-0.2106^a^	-0.2545^a^	-0.1196^a^
		[0.0255]	[0.0317]	[0.0292]
Temperature			-0.0648^a^	-0.0043
			[0.0109]	[0.0082]
Precipitation			0.0018	0.0036^a^
			[0.0022]	[0.0013]
Vegetation			0.0003^a^	0.0001^c^
			[0.0001]	[0.0000]
Night luminosity				2.0503^a^
				[0.3130]
Observations	12,529	12,428	12,356	12,356
R-squared	0.1468	0.1731	0.1987	0.7205
Country FE	Yes	Yes	Yes	Yes
Clustering var.	Grid Cell	Grid Cell	Grid Cell	Grid Cell

The table presents the coefficient estimates of the OLS regression with multiple fixed effects, which examines the association between natural resources and atmospheric particulate matter concentration. We define all variables in Appendix A. In columns (1) to (4), we include country-fixed effects, and all variables are clustered at the grid level. Heteroscedasticity-robust standard errors are reported in parentheses. ^a^, ^b^, and ^c^ indicate statistical significance at the 1%, 5%, and 10% levels (two-tailed), respectively

The relationship remained strongly significant after incorporating geographic and climatic factors into the specification in Columns 2 and 3. In Column 3 (the model which incorporates both geographic and climatic covariates), a unit increase in mineral deposit (onshore petroleum field) was associated with a 0.5932 (0.4314)-point increase in the concentration of particulate matter in a specified grid cell, taking into account geographic and climatic factors.

Moreover, the coefficients of interest remained qualitatively significant at the 1% level even after accounting for a complete set of geo-climatic and economic factors in Column 4. That is, a rise in mineral deposit (onshore petroleum field) within a given 50km grid area is associated with a 0.2583 (0.1349)-point increase in PM level in that area, conditional on a set of geo-climatic and economic characteristics. The findings indicate that the existence of natural resources in a specific area leads to higher levels of particulate matter concentration in that area. Notably, this relationship remained statistically significant at the 1% level across various analytical specifications, highlighting the robustness and consistency of the results.

#### Carbon concentration

Here, we focus on the impact natural resources have on carbon concentration. Inferred from the results presented in Table 5, the unconditional regression demonstrates a positive association between mineral deposits (onshore petroleum field) and carbon concentration in a given area at the 1% level (Column 1). The coefficients of interest suggest that a one-unit increase in mineral deposit (onshore petroleum field) in a given grid cell is associated with a 0.0247 (0.0700)-point increase in atmospheric carbon concentration in that area. After adjusting for geographic effects in Column 2, the coefficient of interest maintained both statistical and economic significance at the 1% level. In Column 3 (controlling for geo-climatic conditions), while the results remained qualitatively unchanged, the significance level shifted to 5% instead. In Column 4, the coefficients of interest showed that a unit increase in mineral deposit (onshore petroleum field) is associated with a 0.0212 (0.0274)-point increase in atmospheric carbon concentration in a given grid cell, conditioned on a complete set of geographic, climatic, and economic factors. The relationship is significant at the 10% and 5% levels, respectively.

**Table 5:** Natural resource and carbon concentration: baseline results

	(1)	(2)	(3)	(4)
Mineral deposit	0.0585^a^	0.0482^a^	0.0287^b^	0.0212^c^
	[0.0131]	[0.0124]	[0.0118]	[0.0119]
Petroleum field	0.0700^a^	0.0495^a^	0.0340^b^	0.0274^b^
	[0.0135]	[0.0137]	[0.0138]	[0.0138]
Latitude		0.0004	0.0000	-0.0003
		[0.0018]	[0.0018]	[0.0018]
Longitude		0.0338^a^	0.0451^a^	0.0446^a^
		[0.0020]	[0.0021]	[0.0021]
Mean elevation		0.0000	0.0002^a^	0.0002^a^
		[0.0000]	[0.0000]	[0.0000]
Ln(distance to coast)		-0.0038	-0.0597^a^	-0.0567^a^
		[0.0059]	[0.0087]	[0.0088]
Temperature			0.0573^a^	0.0586^a^
			[0.0053]	[0.0053]
Precipitation			0.0037^a^	0.0037^a^
			[0.0004]	[0.0004]
Vegetation			0.0001^a^	0.0001^a^
			[0.0000]	[0.0000]
Night luminosity				0.0460^a^
				[0.0069]
Observations	12,529	12,428	12,356	12,356
R-squared	0.3387	0.3532	0.3858	0.3868
Country FE	Yes	Yes	Yes	Yes
Clustering var.	Grid Cell	Grid Cell	Grid Cell	Grid Cell

The table presents the coefficient estimates of the OLS regression with multiple fixed effects, which examines the association between natural resources and carbon concentration. We define all variables in Appendix A. In columns (1) to (4), we include country-fixed effects, and all variables are clustered at the grid level. Heteroscedasticity-robust standard errors are reported in parentheses. ^a^, ^b^, and ^c^ indicate statistical significance at the 1%, 5%, and 10% levels (two-tailed), respectively

### Potential mediating channels

What are the mechanisms underlying the relationship between mineral wealth and environmental health risks? This section investigates several potential proximate channels that could explain the adverse reduced-form association between the existence of natural resources and environmental health risk factors. We hypothesize three sources for these effects (i) the influx of individuals to mineral-rich areas, (ii) activities of extractive companies in these areas, and (iii) the incidence of conflict in resource-endowed regions.

#### The in-migration channel

**Fig. 1:**
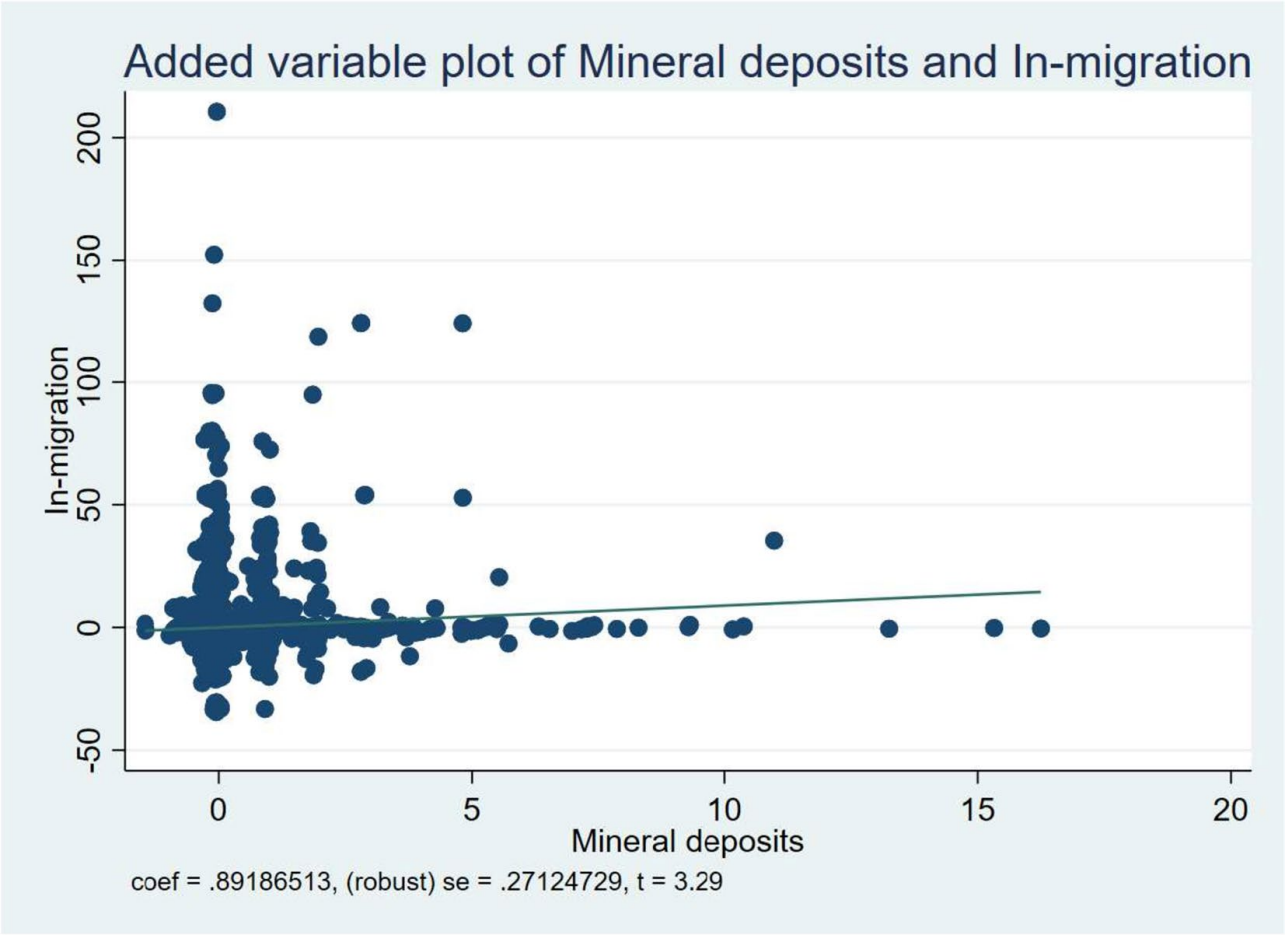
Reduced-form relationship between Mineral deposits and In-migration

One key reason emphasized in our study regarding the negative health outcomes observed in resource-rich regions is the significant influx of people into these areas. Here, we demonstrated that the presence of mineral resources causes an inflow of people (especially job seekers) to these places as presented in Fig. 1, resulting in excessive competition for existing facilities. We present the results of the aforementioned analysis in Table 6.

**Table 6:** Natural resource and environmental health risk- mediating channel of in-migration

	(1)	(2)	(3)	(4)	(5)
	Log Bad water	Log Open defecation	Log Unimproved sanitation	PM	$\boldsymbol{CO}_{\mathbf{2}}$
Mineral deposit:					
Total effect	0.233^a^	0.266^a^	0.304^a^	0.296^a^	0.045^a^
	[0.041]	[0.046]	[0.039]	[0.068]	[0.012]
Direct effect	0.209^a^	0.250^a^	0.283^a^	0.294^a^	0.044^a^
	[0.038 ]	[0.044]	[0.037]	[0.068]	[0.012]
Indirect effect	0.023^a^	0.016^a^	0.022^a^	0.002^b^	0.000
	[0.009]	[0.006]	[0.008]	[0.001]	[0.001]
Main Controls	Yes	Yes	Yes	Yes	Yes
Country FE	Yes	Yes	Yes	Yes	Yes
Clustering var.	Grid cell	Grid cell	Grid cell	Grid cell	Grid cell
Observations	12,356	12,356	12,356	12,356	12,356
Proportion of total effect mediator explains	9.9%	6%	7.1%	0.7%	

The table presents the results of the causal mediating role of in-migration in the relationship between natural resources and environmental health risks. In columns (1) to (5), we include country-fixed effects, and all variables are clustered at the grid level. Heteroscedasticity-robust standard errors are reported in parentheses. ^a^, ^b^, and ^c^ indicate statistical significance at the 1%, 5%, and 10% levels (two-tailed), respectively

Table 6 presents the results of the causal mediating role of in-migration in the relationship between natural resources and environmental health risks. The study presents the findings of estimating five regressions, where each outcome is represented by columns 1 through 5, and the in-migration channel is examined. Just like the baseline models, a set of geo-climatic and economic factors were accounted for. At the 1% significance level, it is observed that the influx of people is a noteworthy mediating factor in how the availability of natural resources affects the reliance on polluted water, open defecation, and unimproved sanitation. On the other hand, at the 5% level, it can be inferred that in-migration plays a substantial mediating role in the relationship between natural resource endowment and particulate matter concentration. More precisely, the findings indicate that in-migration serves as a mediating factor for 9.9%, 6%, 7.1%, and 0.7% in absolute terms, in the causal connection between mineral deposits and the following environmental health risks: dependence on polluted water sources, the practice of open defecation, reliance on unimproved sanitation, and atmospheric particulate concentration; respectively.

#### The extractive industry channel

**Fig. 2:**
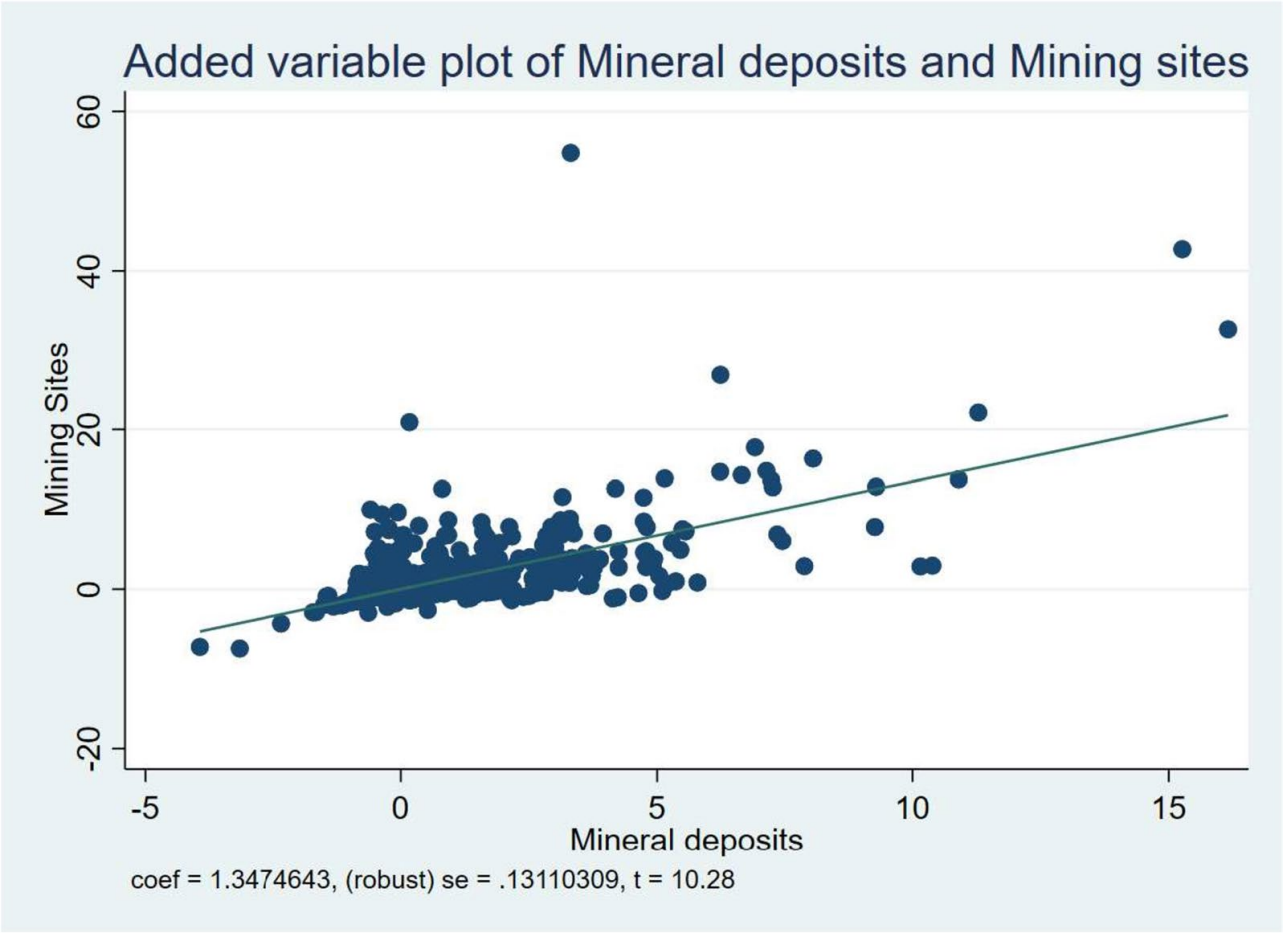
Reduced form Relationship between Mineral deposits and Active mining sites

Another crucial but apparent cause for the reported negative environmental health risk in resource-rich regions is the activity of extractive organizations in these areas. In this context, we utilized the measure of active mining sites from the study conducted by Berman et al. [34] to evaluate the potential mediating influence of extractive industries’ activities. We present the relationship between Mineral deposits and Active mining sites in Fig. 2. The summary of this analysis is presented in Table 7. The structure of the presentation of Table 7 is identical to that of Table 6.

Based on the results presented in Table 7, it can be observed that extractive activities strongly mediates the relationship between the presence of mineral deposits and particulate matter concentration as well as reliance on unimproved sanitation. At the 10% level, it weakly mediates the relationship between mineral deposit and reliance on open defecation The finding also showed that activities of active mining sites played no significant mediating influence on reliance on contaminated water sources and atmospheric carbon concentration. Specifically, activities from mining sites mediates 28.3%, 27.8%, and 43.6% in the absolute terms of the relationship between mineral deposits and reliance on open defecation, unimproved sanitation, and particulate matter concentration; respectively.

**Table 7:** Natural resource and environmental health risk - mediating channel of extractive mining activities

	(1)	(2)	(3)	(4)	(5)
	Log Bad water	Log Open defecation	Log Unimproved sanitation	PM	$\boldsymbol{CO}_{\mathbf{2}}$
Mineral deposit:					
Total effect	0.233^a^	0.266^a^	0.304^a^	0.296^a^	0.045^a^
	[0.041]	[0.046]	[0.039]	[0.068]	[0.012]
Direct effect	0.198^a^	0.190^a^	0.220^a^	0.167^a^	0.035^b^
	[0.050]	[0.053]	[0.044]	[0.059]	[0.015]
Indirect effect	0.035	0.075^c^	0.085^a^	0.129^a^	0.009
	[0.037]	[0.044]	[0.032]	[0.054]	[0.008]
Main Controls	Yes	Yes	Yes	Yes	Yes
Country FE	Yes	Yes	Yes	Yes	Yes
Clustering var.	Grid cell	Grid cell	Grid cell	Grid cell	Grid cell
Observations	12,356	12,356	12,356	12,356	12,356
Proportion of total effect mediator explains		28.3%	27.8%	43.6%	

The table presents the results of the causal mediating role of extractive mining activities in the relationship between natural resources and environmental health risks. In columns (1) to (5), we include country-fixed effects, and all variables are clustered at the grid level. Heteroscedasticity-robust standard errors are reported in parentheses. ^a^, ^b^, and ^c^ indicate statistical significance at the 1%, 5%, and 10% levels (two-tailed), respectively

#### The violent conflict events channel

**Fig. 3:**
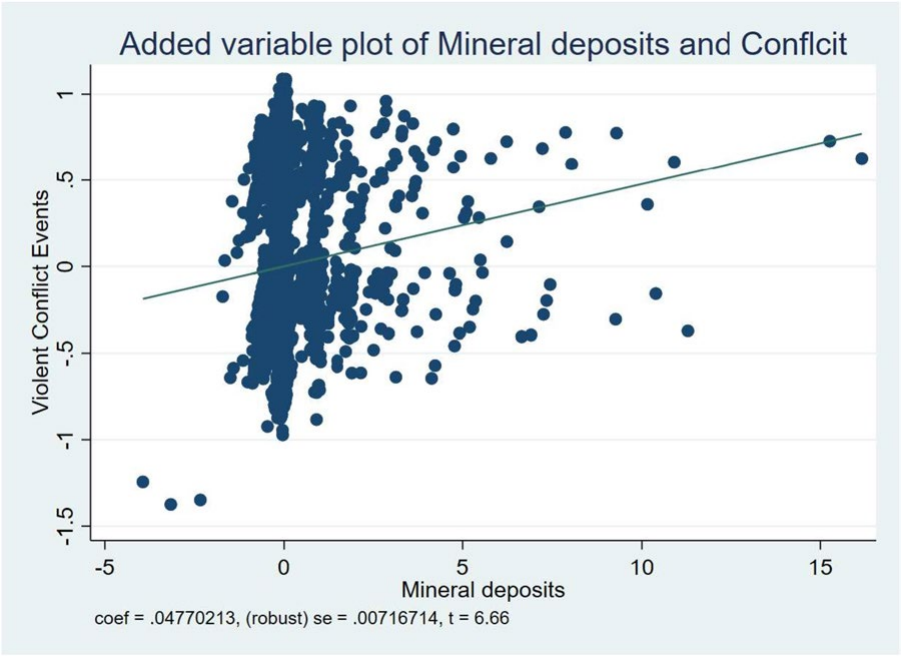
Reduced-form relation between Mineral deposits and Violent Conflict Events

The last potential immediate mechanism resulting in the observed elevated occurrence of environmental hazards in regions abundant with minerals is the frequent presence of significant conflict events in these areas. Similar to prior studies [34, 36, 49], valuable natural resources like minerals are frequently highlighted as a catalyst for armed conflicts. The preceding assertion is highlighted in Fig. 3. These occurrences are usually associated with the loss of life, the destruction of property, and the displacement of people. Table 8 presents the summary of the causal mediating role of violent events in the association between mineral deposits and environmental health risks.

The results presented in Table 8 demonstrate that violent events play a major mediating role in the relationship between mineral deposits and environmental health risks at the 1% and 5% levels. In relation to the influence of mineral deposits on the dependence on substandard facilities, it was observed that violent events mediate 43.8%, 43.4%, and 33.8% of the impact of mineral deposits on reliance on contaminated water sources, open defecation, and unimproved sanitation, respectively. The findings further showed that about 5.5% and 5.2% of the influence of mineral deposits on atmospheric particulate matter and carbon concentration; respectively, is mediated by violent events.

**Table 8:** Natural resource and environmental health risk - mediating channel of violent conflict events

	(1)	(2)	(3)	(4)	(5)
	Log Bad water	Log Open defecation	Log Unimproved sanitation	PM	$\boldsymbol{CO}_{\mathbf{2}}$
Mineral deposit:					
Total effect	0.233^a^	0.266^a^	0.304^a^	0.296^a^	0.045^a^
	[0.041]	[0.046]	[0.039]	[0.068]	[0.012]
Direct effect	0.131^a^	0.150^a^	0.201^a^	0.279^a^	0.042^b^
	[0.036]	[0.039]	[0.034]	[0.066]	[0.012]
Indirect effect	0.102^a^	0.115^a^	0.103^a^	0.0.16^a^	0.002^b^
	[0.013]	[0.015]	[0.013]	[0.003]	[0.001]
Main Controls	Yes	Yes	Yes	Yes	Yes
Country FE	Yes	Yes	Yes	Yes	Yes
Clustering var.	Grid cell	Grid cell	Grid cell	Grid cell	Grid cell
Observations	12,356	12,356	12,356	12,356	12,356
Proportion of total effect mediator explains	43.8%	43.4%	33.8%	5.5%	5.2%

The table presents the results of the causal mediating role of violent conflict events within a grid cell in the documented relationship between natural resources and environmental health risks. In columns (1) to (5), we include country-fixed effects, and all variables are clustered at the grid level. Heteroscedasticity-robust standard errors are reported in parentheses. ^a^, ^b^, and ^c^ indicate statistical significance at the 1%, 5%, and 10% levels (two-tailed), respectively

### Robustness checks

The findings in the preceding subsections support the assertion that natural resources offer significant health and environmental concerns, owing mostly to extractive industries and the inflow of people seeking better economic prospects. Here, we show that the results presented in the study are robust to a large battery of sensitivity checks.

#### Robustness to key infrastructural variables

In Table 9, we delve into the robustness of our findings concerning the influence of mineral deposits on pertinent Environmental Health Risk Factors. In Column 1, where we explore the intricate relationship between mineral deposits and the percentage of the population reliant on contaminated water sources, we introduce two crucial infrastructural variables. These variables encompass the count of pipe boreholes within the respective grid cell, as sourced from Institute for Health Metrics and Evaluation (IHME) [40], and the proximity of the grid cell’s centroid to the nearest water source, as indicated by Wessel and Smith [50]. It is noteworthy that these key variables play an indispensable role in our analysis, potentially underpinning the outcomes we have documented.

Our analytical approach further extends to Column 2, where we investigate the correlation between mineral deposits and open defecation prevalence. Here, we incorporate the count of sewers within the grid cell taken from IHME [40] as a significant variable. Correspondingly, the presence of improved sanitation facilities within the grid cell is incorporated into the examination of mineral deposits’ impact on reliance upon unimproved sanitation. These chosen variables inherently possess the potential to drive the trends we have identified, thus warranting their inclusion in our framework.

Despite potential influences stemming from these key infrastructural variables, it is paramount to underscore the robustness of our outcomes. Specifically, even though the distance to the nearest water body exhibits a notable negative effect on the proportion of individuals relying on contaminated water sources in Column 1, our results retain their robust nature.

**Table 9:** Natural resource and environmental health risk - robustness to key infrastructural variables

	(1)	(2)	(3)
	Log Bad water	Log Open defecation	Log Unimproved sanitation
Mineral deposits	0.1820^a^	0.1899^a^	0.2080^a^
	[0.0306]	[0.0305]	[0.0317]
Petroleum field	0.0690^b^	0.1930^a^	0.1275^a^
	[0.0341]	[0.0366]	[0.0416]
Number of pipe water	-0.0000		
	[0.0000]		
Log distance to water body	-0.4830^a^		
	[0.0286]		
Number of sewers		-0.0000	
		[0.0000]	
Number of sanitation facilities			-0.0000
			[0.0000]
Baseline controls	Yes	Yes	Yes
Observations	12,356	12,356	12,356
R-squared	0.7921	0.7341	0.7714
Country FE	Yes	Yes	Yes
Clustering var.	Grid Cell	Grid Cell	Grid Cell

This table presents the robustness of our baseline findings concerning the influence of mineral deposits on pertinent Environmental Health Risk Factors to key infrastructural variables. Heteroscedasticity-robust standard errors are reported in parentheses. ^a^, ^b^, and ^c^ indicate statistical significance at the 1%, 5%, and 10% levels (two-tailed), respectively

#### Robustness to alternative clustering

In this section of our research paper, we assess the robustness of our findings when subjected to different methods of clustering. Specifically, we explore the impact of conducting two-way clustering on the error terms, considering both grid cell and country levels. Our analysis reveals that our outcomes exhibit robustness across various specifications, with one exception noted in column 5. The results is presented in Table 10.

**Table 10:** Natural resource and environmental health risk - robustness to alternative clustering

	(1)	(2)	(3)	(4)	(5)
	Log Bad water	Log Open defecation	Log Unimproved sanitation	PM	$\boldsymbol{CO}_{\mathbf{2}}$
Mineral deposits	0.1903^a^	0.1905^b^	0.2166^a^	0.2583^a^	0.0212
	[0.0693]	[0.0747]	[0.0591]	[0.0890]	[0.0207]
Petroleum field	0.0436	0.1861	0.1188	0.1349^b^	0.0274
	[0.1171]	[0.1275]	[0.1068]	[0.0669]	[0.0305]
Baseline Controls	Yes	Yes	Yes	Yes	Yes
Country FE	Yes	Yes	Yes	Yes	Yes
Number of Clusters	2	2	2	2	2
Observations	12,356	12,356	12,356	12,356	12,356
R-squared	0.7770	0.7329	0.7670	0.7205	0.3868

This table presents the robustness of our baseline findings concerning the influence of mineral deposits on pertinent Environmental Health Risk Factors to an alternative clustering. Specifically, we explore the impact of conducting two-way clustering on the error terms, considering both grid cell and country levels. Heteroscedasticity clustered standard errors are reported in parentheses. ^a^, ^b^, and ^c^ indicate statistical significance at the 1%, 5%, and 10% levels (two-tailed), respectively

## Discussion

The study found that the presence of these valuable minerals is associated with a heightened prevalence of environmental risks, potentially providing an explanation for the health costs linked to mineral wealth as documented in Von der Goltz and Barnwal [21], Stuckler et al. [51]. The findings of the underlying study provide a comprehensive framework for understanding the multifaceted challenges faced by communities in mineral-rich areas.

The presence of valuable mineral deposits may inadvertently contribute to competition and deterioration in the quality of accessible water sources, thereby exposing local population to various health risks. Reliance on polluted water sources has been increasingly linked to the transmission of diarrhoeal diseases such as cholera, polio, and typhoid [52, 53]. According to the WHO Diarrhoea Report (2023) [54], diarrhoeal diseases is regarded as the second most prominent cause of mortality among children below the age of 5. This could perhaps justify the high prevalence of child-related mortality and morbidity in these areas [55]. The results of the mediation analysis revealed that the heightened dependence on contaminated water sources, as observed, stems from both the influx of individuals into these mineral-rich regions and the occurrence of conflict events. The influx of people to mineral-rich areas may strain the existing clean water infrastructure. With this increased competition, the demand for water may exceed the capacity of available safe water sources, resulting in the use of contaminated or inadequately treated water. Moreover, the findings indicated that violent events are an underlying cause for this observation. As stated earlier, valuable mineral deposits tend to fuel armed fighting [36]. Previous studies have documented the devastating impact conflict events and military actions have on freshwater resources and water infrastructures [56, 57]. Interestingly, our findings unveiled a distinct twist: the noticeable prevalence of depending on contaminated water sources in mineral-rich domains was not propelled by the activities of extractive companies (mines). This finding could be largely attributed to our measurement of mining activities. We relied on the Berman and coauthors measure, which incorporates only large-scale mines [34]. These large-scale extractive organizations often operate under strict regulations and tend to compensate for the environmental repercussions of their actions. Our results corroborate the findings of Dietler et al. [10], which suggest that mining projects could improve household access to modern water infrastructure.

The study further found a positive association between the presence of mineral deposits and reliance on open defecation and unimproved sanitation. Again, this finding clearly underscores the intricate interplay between economic opportunities arising from mineral deposits and the challenges of maintaining essential sanitation infrastructure and practices. Similar findings have been reported by Padhi et al. [58], Leuenberger et al. [59]. Due to the influx of people to these areas, the available sanitation system may be strained, resulting in insufficient access to toilets and waste management services, among other essential hygiene amenities. As a result, the number of people relying on open defecation and inadequate sanitation facilities spikes. Our finding offers an understanding of recent studies that document a negative association between population density and health outcomes [60]. Furthermore, the findings showed that mining activities contribute to the observed adverse effect of mineral deposits and reliance on open defecation and unimproved sanitation. A plausible justification for this observation can be attributed to the enclave nature of these large-scale extractive industries [61]. It is entirely possible that these extractive industries can prioritize their operations over local community development, exacerbating the challenges of poor sanitation. Again, the results underscored that natural resources can, often do, incite and prolong internal conflicts as various armed groups contend for control of the resources or utilize the resources to fund their hostilities. The result of these events is the destruction of lives and properties, making it increasingly difficult to access improved sanitation facilities^6^. This puts stress on existing if not destroyed sanitation facilities, forcing people to rely on unimproved sanitation facilities and practices.

Regarding the relationship between valuable mineral deposits and ambient air quality, the findings showed that at the 1% level, the presence of mineral deposits is positively related to particulate matter concentration. However, a weak association was observed between mineral resources and atmospheric carbon concentration. The results suggest that regions with abundant valuable minerals tend to experience poor air quality, which can have adverse effects on respiratory health and overall well-being. This result offers a clear understanding of the reported prevalence of respiratory health issues in mineral-rich areas [62]. Mining activities can contribute to the release of fine particles into the atmosphere. These particles, often originating from processes like excavation, transportation, and mineral processing, can become suspended in the air, leading to elevated particulate matter concentrations. Also, the sudden surge in people and economic activities in these mineral-rich areas can contribute to the observed increase in particulate matter concentration. This finding corroborates with prior studies [63–65]. The study found that the reported higher carbon concentrations in mineral-rich areas are not caused by extractive industry operations. These observations particularly affirm the assertion that the nature of carbon emissions can be influenced by a range of factors beyond just mining activities, including energy sources, transportation, and land use practices. Moreover, several studies such as Yeboah [66] have demonstrated that large-scale mining enterprises engage in sustainable initiatives, like reforestation, within the regions where their operations are conducted. Convincingly, the robustness analysis shown in Column 5 of Table 10 indicated a diminishing statistical effect of mineral deposits on atmospheric carbon concentration.

The study contributes to a more comprehensive understanding of the environmental challenges posed by mineral wealth, our findings, nevertheless, are not without limitations. First, our analysis relied on cross-sectional study design approach. Although efforts were made to document causal estimates, this approach does not allow us to analyse the relationship over time. Secondly, the measure of activities of extractive companies excludes artisanal mining, hence, our findings should be interpreted in light of large-scale extractive industries. Finally, our documented relationship between mineral deposits and $CO_2$, our mediators, does not explain much of this relationship. In general, albeit our mediators explain about 49.8% to 77.7% of the documented relationship between mineral wealth and environmental health risks, we fail to document all the putative mechanisms driving these intricate relationships.

## Conclusion

This study investigates the environmental health costs associated with the presence of valuable natural resources in Africa. In doing so, the study employed the OLS regression with multiple fixed effects to ascertain this relationship. Our findings unveiled environmental health risks, including reliance on contaminated water sources, open defecation, unimproved sanitation, and poor air quality as the source for the observed adverse health outcomes in mineral-rich regions in Africa. Our findings underscore the importance of implementing stringent environmental regulations and monitoring mechanisms to mitigate the potential negative impact of natural resource abundance/dependence.

The findings somewhat affirm the enclave nature as the economic benefits brought about by mining activities might not always be accompanied by proportional investments in sanitation infrastructure, resulting in increased reliance on unimproved structures. The study suggests that the sudden surge in population for better economic opportunities, activities of extractive companies, and the potential of conflict in a mineral-rich environment are the rooted cause of the incidence of environmental health risks.

### Supplementary Information

**Additional file 1.** The Online Appendix file provides a detailed account of the data utilised for the underlying study and its sources, descriptive statistics for our study’s variables, and a sample of the constructed 50km grid borders for the entire African continent.

## Data Availability

In the present study, secondary data was used. Syntax and data supporting the conclusions of this article are available in the Open Science Framework Repository [68].

## Footnotes

1 Africa mining iQ [3] “Africa’s mineral industry leads globally with diverse reserves, including gold, bauxite, cobalt, and platinum-group metals, fostering economic growth and development. The continent boasts a substantial number of mining projects, totaling 2039 across its various regions.”

2 Mineralized urbanization is defined as the catalysing effect of mineral discovery, production cycles, and commodity chains on urban growth and settlement patterns at local, regional, and national levels, in the context of oscillating booms and busts of global mineral markets and eventual mineral depletion [28]

3 These estimates were produced using a geo-positioned dataset created from 634 household surveys. Survey sources used include the Demographic and Health Survey (DHS) and UNICEF Multiple Indicator Cluster Survey (MICS) series and other country-specific surveys.

4 The critical minerals covered in this dataset are Antimony (Sb), Barite (Barium, Ba), Beryllium (Be), Cobalt (Co), Fluorite or Fluorspar (Fluorine, F), Gallium (Ga), Germanium (Ge), Graphite (Carbon, C), Hafnium (Hf), Indium (In), Lithium (Li), Manganese (Mn), Niobium (Nb), Platinum-Group Elements (PGE), Rare-Earth Elements (REE), and Rhenium (Re)

5 Figure A1 presents our constructed grid cell with the mineral deposits plotted on it in Appendix A.2

6 Collier and Sambanis [35] described conflict trap as a phenomenon that hampers development and escalates the potential for conflict, thus complicating the distribution of crucial public infrastructure in regions susceptible to conflict.
