# Supplementary material for: Mineral wealth paradox: health challenges and environmental risks in African resource-rich areas
Source: BMC Public Health. 2024 Mar 6;24:724. doi: 10.1186/s12889-024-18137-1 (PMC11334305; doi:10.1186/s12889-024-18137-1)
Supplement: Supplementary file 1 — Additional file 1. The Online Appendix file provides a detailed account of the data utilised for the underlying study and its sources, descriptive statistics for our study’s variables, and a sample of the constructed 50km grid borders for the entire African continent. [file 12889_2024_18137_MOESM1_ESM.pdf]

Mineral Wealth Paradox: Health Challenges and Environmental Risks in African Resource-Rich Areas

Online Appendix

Emmanuel Adu Sarfo[1, 2] and Rabbi Tweneboah[1*]

[1]Faculty of Economic Sciences, National Research University, Higher School of Economics, 20 Myasnitskaya Street, 101000, Moscow, Russia.
[2]Department of Mathematics and Statistics, Youngstown State University, 1 University Plaza, OH 44555, Ohio, United States.

*Corresponding author(s). E-mail(s): rtveneboakh@edu.hse.ru;
Contributing authors: eadusarfo@edu.hse.ru;

Appendix A

A.1 Variable Definition and Source

1. Log bad water: The percentage of the population without access to a specific type of drinking water source at each 5x5 km pixel. Source: Institute for Health Metrics and Evaluation (IHME) [1].
2. Log open defecation and Log unimproved sanitation: Reliance on Open defecation and other Unimproved Sanitation represents the percentage of the population without access to a specific type of sanitation facility at each 5x5 km pixel. Source: Institute for Health Metrics and Evaluation (IHME) [1].
3. Particulate Matter (PM2.5) Concentration: This represents the average concentration of fine particulate matter (PM2.5) present in the ambient air. PM2.5 refers to particles suspended in the air with a diameter of 2.5 micrometers or smaller, which can pose health risks when inhaled due to their ability to penetrate deep into the respiratory system. This variable is of critical importance in understanding the impact of ambient air pollution on global health. Data is available from 1990 to 2013 and is sourced from Brauer et al. [2].
4. CO2: Represents the mean concentration of carbon dioxide (CO2) contained within a vertical column of dry air that extends from the Earth's surface up to the outer boundary of the atmosphere. This concentration measurement is typically expressed in parts per million (ppm) by volume. The data used for this variable are derived from NASA's Orbiting Carbon Observatory-2 (OCO-2) project. The provided raster dataset captures this CO2 concentration by utilizing a 10-kilometer grid. Source: OCO-2 Science Team/Michael Gunson, Annmarie Eldering [3].
5. Mineral deposits: Mineral Deposits in Africa pertains to the spatial distribution of mineral resource deposits across the African continent. These mineral deposits encompass 22 specific minerals or mineral commodities that are considered critical for the economic and security interests of the United States, as of the year 2017. The dataset consists of both point and polygon layers within a geodatabase from Schulz et al. [4].
6. Petroleum field: The Petroleum Dataset (PETRODATA) is a comprehensive collection of global oil and gas fields. This dataset is specifically curated to provide detailed information about the geographic distribution of hydrocarbon reserves around the world. It is tailored for utilization within geographic information systems (GIS), offering a specialized resource for visualization, manipulation, and in-depth analysis. Within this dataset, essential details about oil and gas fields are meticulously documented. The data is taken from Lujala et al. [5].
7. In-Migration: Estimated internal human migration flows between subnational administrative units for malaria-endemic countries. This data captures a 5year internal human migration flow (number of people) between 2005 and 2010. For each country, it also included a GHA_AdminUnit_Centroids

shapefile representing the subnational administrative units of origin and destination Source: World Health Organization [6]

8. Violent Conflict Events: We make use of the Armed Conflict Location and Event dataset [7], which provides data regarding the geographical locations of conflict incidents across African nations. These incidents are sourced from a variety of outlets, such as regional and local news reports, humanitarian organizations, and scholarly publications. One distinct aspect of ACLED is its inclusive documentation of all forms of political violence, encompassing acts against civilians, riots, and protests both within and beyond civil conflicts, without necessitating a minimum threshold for battle-related fatalities. Additionally, ACLED provides insights into event categories, their outcomes, and the attributes of involved parties on both opposing sides of the conflict.
9. Berman Mineral: The Raw Material Data (RMD) dataset primarily gathers data pertaining to large-scale mines, typically managed by multinational corporations or governmental bodies. Consequently, our sample does not encompass small-scale mines or those that operate outside the bounds of legality. This data was borrowed from Berman et al. [8]
10. Mean elevation: Global elevation (in meters) from Shuttle Radar Topography Mission (SRTM) dataset (v4.1) at 500m resolution.
11. Temperature: Average monthly air temperature per year in degrees Celsius. Created using UDel Air Temperature dataset.
12. Precipitation: Total precipitation per year in millimeters. Created using UDel Precipitation dataset.

Table A1 Summary Statistics of Baseline Variables

	Sample size	Mean	Standard deviation	Minimum	Maximum
Log bad water	12529	4.752	3.732	0.000	12.425
Log open defecation	12529	5.133	3.686	0.000	12.009
Log unimproved sanitation	12529	5.037	3.664	0.000	12.416
Particulate Matter (PM2.5) Concentration	12529	0.344	1.832	0.000	52.077
CO2	12529	402.432	0.917	399.491	406.474
Mineral deposits	12529	0.095	0.587	0.000	17.000
Petroleum field	12529	0.166	0.541	0.000	5.000
Latitude	12529	18.600	16.053	-25.359	63.574
Longitude	12529	6.057	17.306	-34.823	37.657
Mean elevation	12428	611.082	436.968	-76.385	2843.352
Temperature	12360	24.026	3.468	8.864	30.234
Precipitation	12360	56.927	51.291	0.000	259.162
Vegetation	12529	3253.490	1883.494	71.344	7544.809
Night luminosity	12529	0.087	0.694	0.000	31.292
Log distance to coast	12529	12.650	2.094	0.000	14.403

A.2 Constructed 50km × 50km Africa grid cell

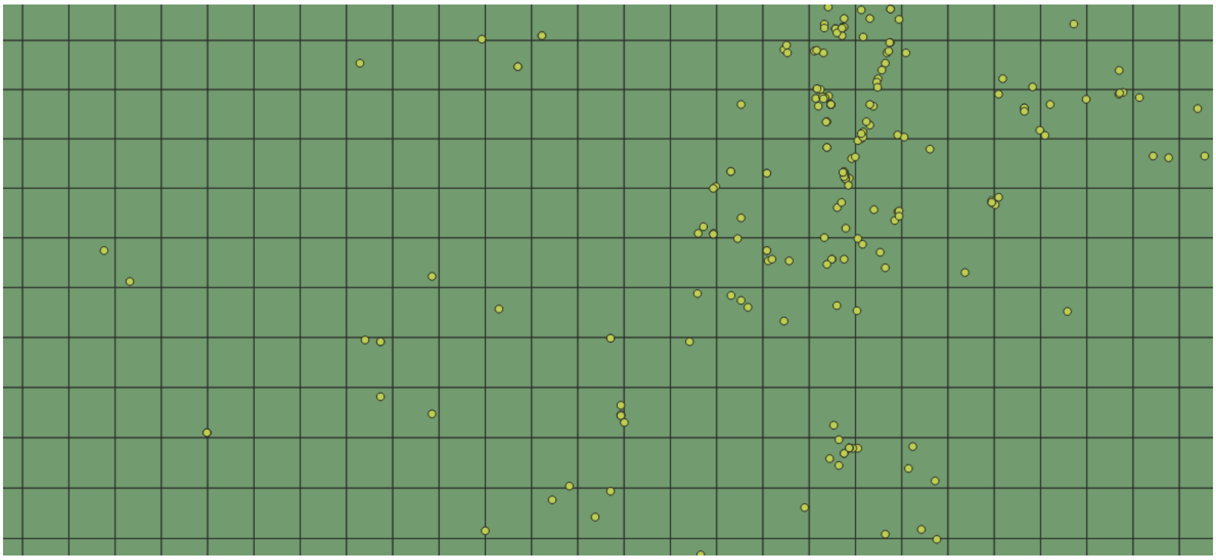

Fig. A1 This figure shows a snapshot of our constructed 50km × 50km grid cell with the locations of mineral deposits from [4] plotted on it.

References

[1] Institute for Health Metrics and Evaluation (IHME).: Low- and Middle-Income Drinking Country Water and Sanitation Facilities Access Geospatial Estimates 2000-2017. Institute for Health Metrics and Evaluation (IHME). Available from: http://ghdx.healthdata.org/record/ihme-data/lmic-wash-access-geospatial-estimates-2000-2017.

[2] Brauer M, Freedman G, Frostad J, Van Donkelaar A, Martin R, Dentener F, et al. Ambient Air Pollution Exposure Estimation for the Global Burden of Disease 2013. Environmental Science and Technology. 2015;50(1):79–88. https://doi.org/10.1021/es5053834.

[3] OCO-2 Science Team/Michael Gunson, Annmarie Eldering.: OCO-2 Level 2 bias-corrected XCO2 and other select fields from the full-physics retrieval aggregated as daily files, Retrospective processing V10r. Greenbelt, MD, USA. Accessed: 2021-07-20. Goddard Earth Sciences Data and Information Services Center (GES DISC).

[4] Schulz KJ, DeYoung Jr JH, Seal II RR, Bradley DC. Critical mineral resources of the United States: Economic and environmental geology and prospects for future supply. 1802. U.S. Geological Survey; 2017. Available from: https://doi.org/10.3133/pp1802.

[5] Lujala P, Rø d JK, Thieme N. Fighting over Oil: Introducing A New Dataset. Conflict Management and Peace Science. 2007;24(3):239–256. https://doi.org/10.1080/07388940701436469.

[6] World Health Organization.: World malaria report. https://apps.who.int/iris/bitstream/handle/10665/200018/9789241565158_eng.pdf;jsessionid=ED89189FD617028F30DDF55459BF77C3?sequence=1.

[7] Raleigh C, Linke A, Dowd C. Armed Conflict Location and Event Data Project (ACLED) Codebook Version 3. Oslo: Peace Research Institute Oslo; 2014. Available from: http://www.acleddata.com/data/acled-versions-1-6-data-1997-2015/.

[8] Berman N, Couttenier M, Rohner D, Thoenig M. This mine is mine! How minerals fuel conflicts in Africa. American Economic Review. 2017;107(6):1564–1610.
